# Association between Anemia and Risk of Parkinson Disease

**DOI:** 10.1155/2021/8360627

**Published:** 2021-07-07

**Authors:** Yao-Chin Wang, Abel Po-Hao Huang, Sheng-Po Yuan, Chu-Ya Huang, Chieh-Chen Wu, Tahmina Nasrin Poly, Suleman Atique, Woon-Man Kung

**Affiliations:** ^1^Department of Emergency, Min-Sheng General Hospital, Taoyuan, Taiwan; ^2^Graduate Institute of Injury Prevention and Control, College of Public Health, Taipei Medical University, Taipei, Taiwan; ^3^Division of Neurosurgery, Department of Surgery, National Taiwan University Hospital, Taipei, Taiwan; ^4^Graduate Institute of Biomedical Informatics, College of Medical Science and Technology, Taipei Medical University, Taipei, Taiwan; ^5^Department of Otorhinolaryngology, Wan Fang Hospital, Taipei Medical University, Taipei, Taiwan; ^6^Department of Otorhinolaryngology, Shuang-Ho Hospital, Taipei Medical University, New Taipei City, Taiwan; ^7^Taiwan College of Healthcare Executives, Taipei, Taiwan; ^8^Department of Exercise and Health Promotion, College of Kinesiology and Health, Chinese Culture University, Taipei, Taiwan; ^9^Department of Health Informatics, College of Public Health and Health Informatics, University of Ha'il, Ha'il, Saudi Arabia

## Abstract

**Methods:**

We systematically searched articles on electronic databases such as PubMed, Embase, Scopus, and Google Scholar between January 1, 2000 and July 30, 2020. Articles were independently evaluated by two authors. We included observational studies (case-control and cohort) and calculated the risk ratios (RRs) for associated with anemia and PD. Heterogeneity among the studies was assessed using the *Q* and *I*^2^ statistic. We utilized the random-effect model to calculate the overall RR with 95% CI.

**Results:**

A total of 342 articles were identified in the initial searches, and 7 full-text articles were evaluated for eligibility. Three articles were further excluded for prespecified reasons including insufficient data and duplications, and 4 articles were included in our systematic review and meta-analysis. A random effect model meta-analysis of all 4 studies showed no increased risk of PD in patients with anemia (*N* = 4, RR_adjusted_ = 1.17 (95% CI: 0.94-1.45, *p* = 0.15). However, heterogeneity among the studies was significant (*I*^2^ = 92.60, *p* = <0.0001). The pooled relative risk of PD in female patients with anemia was higher (*N* = 3, RR_adjusted_ = 1.14 (95% CI: 0.83-1.57, *p* = 0.40) as compared to male patients with anemia (*N* = 3, RR_adjusted_ = 1.09 (95% CI: 0.83-1.42, *p* = 0.51).

**Conclusion:**

This is the first meta-analysis that shows that anemia is associated with higher risk of PD when compared with patients without anemia. However, more studies are warranted to evaluate the risk of PD among patients with anemia.

## 1. Introduction

### 1.1. Rationale

Parkinson disease (PD) is the second most common age-related neurodegenerative disorder, with more than 60,000 newly diagnosed cases yearly in the USA [1]. The incidence of PD has been increasing at an alarming rate and is estimated to be nearly 1.2 million PD cases worldwide by 2030 and 12 million patients worldwide by about 2050 [2]. It is estimated that both direct and indirect costs regarding PD are approximately USD 52 billion only in the USA [2]. The higher amount of financial burden that places on our current health care system highlights the importance of conducting research to curb the incidence and prevalence rate of PD. Therefore, a vivid understanding of PD risk factors can help researchers and policymaker to develop strong strategies for turning down the number of new cases and reducing costs.

The common risk factors of developing PD are exposure to pesticides, genetic variants, environment toxins, idiopathic REM sleep behavior disorder (RBD), and focal cerebrovascular damage [3–6]. However, several epidemiological studies have reported that inflammatory bowel disease (IBD) [7], head injury [8], and autoimmune rheumatic diseases (ARDs) [9] are significantly associated with increased risk of PD. An increased risk of PD was also reported in patients with anemia than those without anemia. Although the exact relationship between anemia and PD risk remains inconclusive, several possible hypotheses have been proposed. A recent study [10] reported that chronic anemia can increase brain hypoxia which is the main risk factor of Alzheimer disease (AD). Previous studies also suggested that patient with AD may experience sequela of epigenetic changes during the development stage [11, 12]. A study showed that neonatal iron deficiency is associated with modification of the AD-related gene expression [13]. Since AD is associated with PD [14], these etiology and link may lead to increased risk of PD. Furthermore, another study demonstrated that rats with iron deficiency diet reduced dopaminergic activity which might induce PD risk [15].

Anemia hampers erythropoiesis and enhances eryptosis, whereas insufficient iron is reported in the substantia nigra of PD patients [16, 17]. Increased serum iron levels are related to decreased risk of PD, and high dietary iron intake reduces PD risk [18, 19]. Moreover, dysregulation of iron metabolism is associated with oxidative stress and cell death [20–22]. Based on biological and epidemiological evidence, it is needed to conduct a study that summarizes the role of anemia on PD risk. However, to our best knowledge, no meta-analysis has been performed to assess the magnitude of association between anemia and PD.

*Goal:* The aim of this current systematic review and meta-analysis is to evaluate the published epidemiological studies for clarifying the association between anemia and risk of PD.

### 1.2. Research Questions

- Study the magnitude of the risk of PD in patients with anemia and without anemia
- Calculate the magnitude difference of the risk of PD in male and female patients with anemia
- Reduce the confounding factors by evaluating the risk based on various adjustments

## 2. Methods

### 2.1. Search Strategy

We did a systematic search on electronic databases such as PubMed, Embase, Scopus, and Web of Science between January 1, 2000 and July 30, 2020. The following search terms were used to collect relevant articles: “anemia,” OR “low level hemoglobin,” “iron deficiency” And “Parkinson disease.” The initial search was conducted by one author who is an expert in systematic review. Moreover, we searched in the reference lists of retrieved articles to ensure the comprehensiveness.

### 2.2. Eligibility Criteria

The eligibility criteria were restricted to observational studies (case-control and cohort) and clinical trials (randomized control trial) that evaluated the association between anemia and PD as a primary outcome. Studies were included if they were published in the form of (a) original study, (b) participants at least 200, (c) published in English, (d) provided clear definition of anemia and PD, and (e) provide proper effect size to summarized pool risk.

We excluded studies if they published in the form of review, short report, poster, editorial, case-report, and correspondence.

### 2.3. Selection Process

Two authors (TNP and YCW) independently reviewed all the titles and abstracts of retrieved articles. They used prespecified selection criteria to select relevant articles to be included in this meta-analysis. Any disagreement in this stage was resolved by the difference by discussing with third author.

### 2.4. Data Extraction

Same two authors developed data collection form for extracting the required data from the selected studies. They checked the study duplication by comparing authors' names, publication year, and location of study. Two authors collected the information about effect size (odds ratios, hazard ratios with 95% CI), adjusted factors, total number of participants, number of anemia and PD patients, inclusion and exclusion criteria of anemia and PD, age, percentage of male, duration of study period, and location.

### 2.5. Risk Assessment

The Newcastle-Ottawa Scale (NOS) was used to assess the quality of each study. They calculated the NOS score and divided into two groups: low and high qualities. The heterogeneity was calculated among study-specific RRs using the *Q* and *I*^2^ statistic. Finally, publication bias evaluated by 3 funnel plot–based methods: the Egger's test, the Begg's test, and the trim and fill method.

### 2.6. Statistical Analysis

The meta-analysis was performed on selected studies that evaluated the magnitude of the association by using adjusted ORs and HRs. The pooled risk ratio (RR) was calculated from selected studies to show the effect size. The random-effect model was used in this meta-analysis. We calculated statistical heterogeneity across the various studies which were tested using the Cochran *Q* statistic and quantified by the *I*^2^ value. The heterogeneity among the studies was categorized into four groups, namely, very low (<25%), low (25~50%), medium (50~75%), and high (>75%) [23–25]. When number of studies is small, the random-effect model is a perfect test to reduce bias and heterogeneity among the studies. We draw forest plot to present effect size and funnel plot to depict the publication bias. However, all the statistical analyses were performed using comprehensive meta-analysis software (V:2). The *p* value less than 0.5 is considered as a significant.

## 3. Results

### 3.1. Study Selection

**Figure 1:**
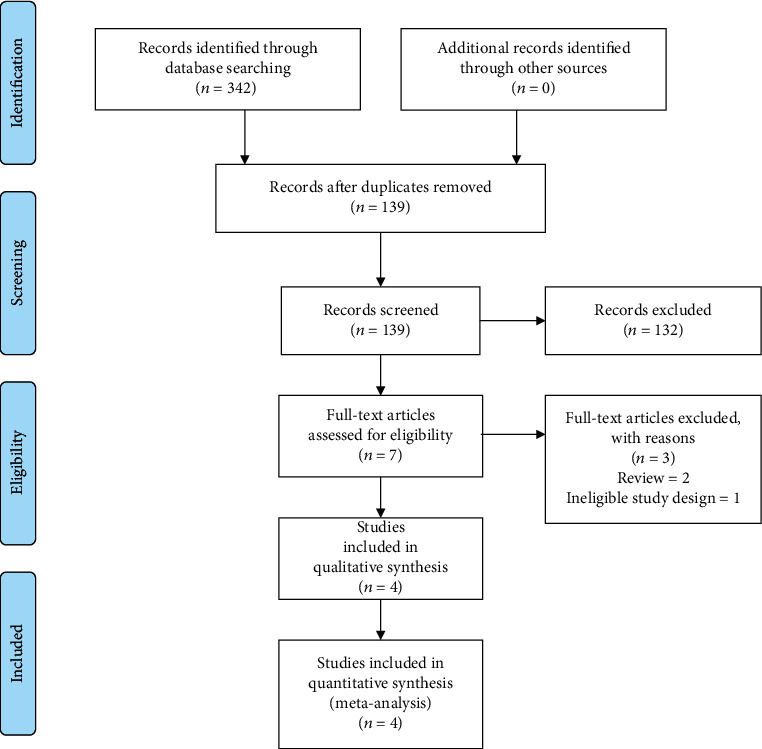
Flow diagram of the study selection process.

Initial search in the electronic databases yielded 342 articles. A total of 335 articles were excluded after reviewing the titles and abstracts of retrieved articles. However, 7 articles went to full-text review, and 3 articles were further excluded due to not matching the prespecified selection criteria. Finally, 4 articles were included in the current systematic review and meta-analysis [26–29].  Figure 1 shows our study selection process.

### 3.2. Study Characteristics

This current systematic review and meta-analysis comprised of 4 studies including 3 cohorts and 1 case-control study (Table 1). Included studies published between 2009 and 2019; the average age of patients was from 48.7 to 71. The percentage of male patients was from 24.1 to 63%. Three studies followed World Health Organization (WHO) guidelines to be included in the anemia patients: hemoglobin level < 13 g/dL for men and <12 g/dL for women, and one study used ICD to include anemia patients. On the other hand, three studies used ICD to include PD patients, and one study used PD registry linkage.

### 3.3. Anemia and Risk of PD

**Figure 2:**
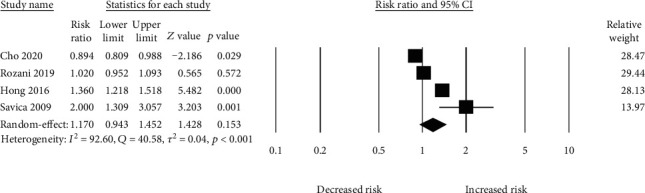
Association between anemia and risk of PD.

There were various follow-up durations for each of the 4 studies, ranging from 5 to 8.8 years. Our meta-analysis comprised a total of 92,851 PD patients. A random-effect model meta-analysis of all 4 studies showed no increased risk of PD in patients with anemia (*N* = 4, RR_adjusted_ = 1.17 (95% CI: 0.94-1.45, *p* = 0.15). However, heterogeneity among the studies was significant (*Q* = 40.58, tau^2^ = 0.04, *I*^2^ = 92.60, *p* = <0.0001) (Figure 2).

### 3.4. Subgroup Analysis

**Figure 3:**
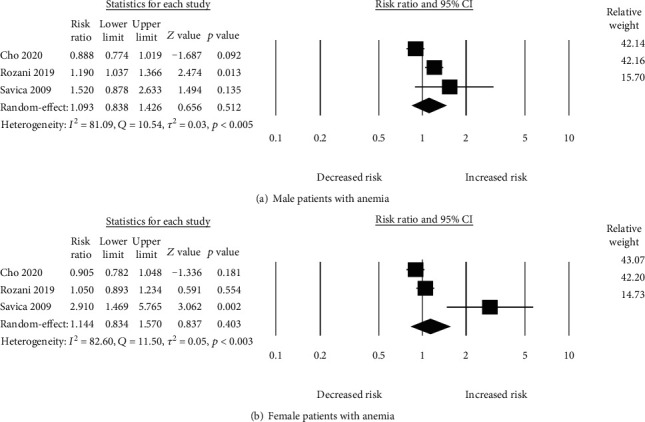
Risk of PD.

We pooled 3 studies in order to evaluate the risk of PD based on gender. The pooled relative risk of PD in female patients with anemia was higher (*N* = 3, RR_adjusted_ = 1.14 (95% CI: 0.83-1.57, *p* = 0.40) as compared to male patients with anemia (*N* = 3, RR_adjusted_ = 1.09 (95% CI: 0.83-1.42, *p* = 0.51) (Figure 3). However, a significant heterogeneity was both in male (*I*^2^ = 81.02, *p* = 0.005, *Q* = 10.54, tau^2^ = 0.03) and female (*I*^2^ = 82.60, *p* = 0.003, *Q* = 11.50, tau^2^ = 0.05) patients with anemia.

We also evaluated the risk of PD in patients among the studies adjusted with most common confounding factors such as diabetes and hypertension. The pooled risk ratio was (*N* = 3, RR_adjusted with diabetes and hypertension_ = 1.10 (95% CI: 0.73-1.66, *p* = 0.64), and heterogeneity was significant (*I*^2^ = 96.72, *p* = <0.001, *Q* = 30.49, tau^2^ = 0.08).

### 3.5. Publication Bias

**Figure 4:**
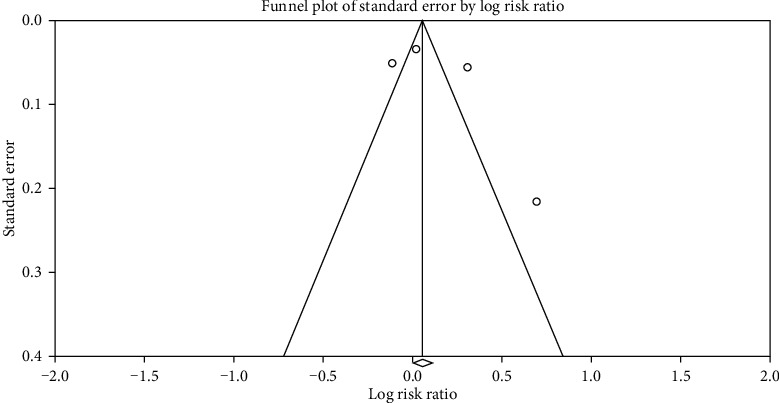
Funnel plot.

Figure 4 shows the funnel plot indicating possible publication bias. Egger's regression test of the funnel asymmetry presented no possible publication bias.

## 4. Discussion

### 4.1. Main Findings

To our knowledge, this is the first study to assess the risk of PD among the patients with anemia. This current meta-analysis of four observational studies showed that patients with anemia were not significantly associated with 17% increased risk of PD as compared with nonanemia. However, male patients with anemia showed nonsignificant increased risk of PD while comparing with female patients with anemia. The strengths of this study are (a) all included studies quality were high and (b) consider adjusted effect size; therefore, risk of bias is low and (c) clear definition of anemia and PD risk.

### 4.2. Biological Mechanism

The biological mechanism of PD and anemia is not fully understood yet. However, there are several biological explanations that can define their association. First, the neural degeneration and disease progression among the PD patients can be induced by either apoptosis or necrosis [30]. The brain cell death is caused by DNA fragmentation and typical morphological changes including cell shrinkage and nuclear condensation. However, apoptosis in PD is still debatable. The animals and in vitro studies reported apoptosis as well as promoting the rate of neurodegeneration [31–33]. Second, some assumptions regarding oxidative stress and iron metabolism are likely to dopaminergic neural death in the central nervous system [34, 35]. Several studies analyzed erythrocytes of PD indicating lessened superoxide dismutase, glutathione peroxidase activity, and elevated lipid peroxidation which are responsible for the degeneration of dopaminergic neurons in the substantia nigra. All the process, however, are related to the activity of oxidative stress [36, 37]. Third, *α*-synuclein and etiology of PD are the main keys that can be used to explain the pathological mechanism of anemia and PD risk. Erythrocytes generate maximum portion of *α*-synuclein in the human blood [38]; it has shown in the previous study that the ratio of *α*-synuclein oligomer and total protein is higher in erythrocyte of PD patients when compared to normal patients [39]. Anxiety and depression are most common symptoms in PD that are considered as primary contributors to abnormality, low quality of life, and low survival [40, 41]. Depression in PD patient is associated with several neurotransmitter dysfunctions including serotonin, noradrenaline, and dopamine [42]. Moreover, gut brain axis (GBA) has a positive correlation with increased risk of PD. Accumulation of gut microbiota in *α*-synuclein in PD has received wide attention over the past years. A previous mice study showed that microbial metabolites may increase the risk of neuroinflammation by expressing proinflammatory cytokines which ultimately lead to the development of motor symptom [43]. Other study also reported a positive link of gut microbiota in neurodegeneration [44].

### 4.3. Clinical Implications

PD is now one of the leading causes of disability and deaths globally. A significant amount of epidemiological studies have highlighted an increased prevalence of PD which raised sheer concern and urgent need for public health strategies [45–47]. Appropriate planning would help to reduce the number of PD patients as well as healthcare cost over the coming decades [48]. The primary risk factor of PD is age, but PD also appears to be linked to anemia. However, the association between anemia and PD based on duration and severity is less well known. Since anemia and aging population are increasing globally, the prevalence of PD undoubtedly will increase. Conducting a meta-analysis can clarify the effect size in order to prevent and treat the disease both earlier and effectively.

### 4.4. Strengths and Limitations

Our first and rigorous meta-analysis has several strengths. First, this is the first meta-analysis that investigated the association between anemia and PD risk. Second, this study included four large observation studies from Taiwan, Israel, South Korea, and USA; healthcare data quality of these countries is world standard. They adjusted potential confounding factors to this study to reduce bias of effect size calculation. Third, this present study showed risk difference between male and female patients.

However, this study has some limitations. First, number of included studies is only four, although all are high quality study design with larger number of participants. Second, we were not able to provide risk of PD based on severity of anemia (high, moderate, and low) and BMI (overweight, obese, and underweight). Third, the pooled risk was not calculated based on location, study design, and other factors like smoking status, alcohol consumptions, and duration of anemia; it is because data were limited.

## 5. Conclusion

Our study shows that the risk of PD was higher among the patients with anemia as compared without anemia. The risk of PD was higher in male patients. Therefore, more protective strategies should be taken in male patients when compared to female patients. The risk of PD among patients with anemia can be explained by some biological mechanisms like oxidative stress and downregulation of iron homeostasis. More observational studies in different regions and biological studies are warranted to clarify the mechanism underlying their association.

## Figures and Tables

**Table 1:** Baseline characteristics of included studies.

Author name	Location	Study duration	Age	Male (%)	Design	PD	Anemia criteria	PD	Mean follow-up (Yrs.)	HR/OR	Major adjustment	NOS score
Cho 2020	S. Korea	2009-2013	57.41 ± 7.51	63	Cohort	3,844	The World Health Organization (WHO): hemoglobin level < 13 g/dL for men and <12 g/dL for women	ICD-10	5	0.89 (0.80-0.98)	Smoking, alcohol, physical activity, HTN, DM, dyslipidemia, GFR	8
Rozani 2019	Israel	1999-2012	48.7	47.4	Cohort	2,427	The World Health Organization (WHO): hemoglobin level < 13 g/dL for men and <12 g/dL for women	ICD-9	8.8 ± 3.9	1.02 (0.95-1.09)	N/A	8
Hong 2016	Taiwan	N/A	56.4	24.1	Cohort	86,334	ICD-9	ICD-9	6.6	1.36 (1.22-1.52)	HTN, DM, hyperlipidemia	8
Savica 2009	USA	1976-1995	71	61.7	Case-control	196	The World Health Organization (WHO): hemoglobin level < 13 g/dL for men and <12 g/dL for women	Records linkage system	n/a	2.00 (1.31-3.06)	N/A	8

#PD: Parkinson disease; ICD: International Classification of Diseases; HR: hazard ratio; OR: odds ratio; NOS: the Newcastle-Ottawa Scale.

## Data Availability

The data used to support the findings of this study are from previously reported studies and datasets, which have been cited and included within the article.
